# Optogenetically controlled human functional motor endplate for testing botulinum neurotoxins

**DOI:** 10.1186/s13287-021-02665-3

**Published:** 2021-12-05

**Authors:** Juliette Duchesne de Lamotte, Jérôme Polentes, Florine Roussange, Léa Lesueur, Pauline Feurgard, Anselme Perrier, Camille Nicoleau, Cécile Martinat

**Affiliations:** 1 IPSEN Innovation, 5 avenue du Canada, 91940 Les Ulis, France; 2 Université Evry-Paris Saclay/INSERM UMR861, Institut Des Cellules Souches Pour Le Traitement Et L’étude Des Maladies Monogéniques (I-Stem), 2 rue Henri Auguste Desbruères, 91100 Corbeil-Essonne, France; 3 Laboratoire Des Maladies Neurodégénératives: Mécanismes, thérapies, imagerie, Université Paris Saclay/CEA/CNRS UMR9199, MIRCen, Bâtiment 61, CEA-Fontenay-Aux-Roses, 18 route du Panorama, 92265 Fontenay-aux-Roses, France

**Keywords:** Human-induced pluripotent stem cells, Motor endplate, Functional, Botulinum neurotoxins, Calcium indicators, Optogenetics

## Abstract

**Background:**

The lack of physiologically relevant and predictive cell-based assays is one of the major obstacles for testing and developing botulinum neurotoxins (BoNTs) therapeutics. Human-induced pluripotent stem cells (hiPSCs)-derivatives now offer the opportunity to improve the relevance of cellular models and thus the translational value of preclinical data.

**Methods:**

We investigated the potential of hiPSC-derived motor neurons (hMNs) optical stimulation combined with calcium imaging in cocultured muscle cells activity to investigate BoNT-sensitivity of an in vitro model of human muscle-nerve system.

**Results:**

Functional muscle-nerve coculture system was developed using hMNs and human immortalized skeletal muscle cells. Our results demonstrated that hMNs can innervate myotubes and induce contractions and calcium transient in muscle cells, generating an in vitro human motor endplate showing dose-dependent sensitivity to BoNTs intoxication. The implementation of optogenetics combined with live calcium imaging allows to monitor the impact of BoNTs intoxication on synaptic transmission in human motor endplate model.

**Conclusions:**

Altogether, our findings demonstrate the promise of optogenetically hiPSC-derived controlled muscle-nerve system for pharmaceutical BoNTs testing and development.

**Supplementary Information:**

The online version contains supplementary material available at 10.1186/s13287-021-02665-3.

## Background

Botulinum neurotoxins (BoNTs), produced by various *Clostridium* bacteria, are among the most potent neurotoxins known, and exposure induces flaccid muscular paralysis [1–4]. Since the 1980s, despite their toxicity and because small doses produce a prolonged action at the synapses, BoNTs have been approved for therapy of many neuromuscular and neurological disorders characterized by excessive muscle tone or abnormal muscle contractions [5]. Based upon the use of neutralizing antibodies, at least seven different serotypes of BoNT have been reported, from BoNT/A to BoNT/G, and recent genomic sequencing approaches are likely to reveal more. Serotypes differ in their toxicity, molecular site of action, efficiency in terms of muscle paralysis, duration of action and specific affinity for their targets [6–11]. Although each serotype has its particular characteristics, at the neuromuscular junction (NMJ), they all induce an inhibition of the ACh release into the synaptic cleft, leading to denervation and weakening of muscle contractions.

Despite the great diversity of natural BoNTs, only serotypes A and B have reached the market so far. Currently, there are four formulations of BoNTs approved by the US Food and Drug Administration (FDA) for several clinical or aesthetics indications [12, 13]. Several dozen clinical trials are underway to study the efficacy and safety of BoNTs for various clinical conditions [13]. The increasing understanding of the biology of the neurotoxins and the availability of highly differentiated toxin serotypes as well as engineered serotypes offers the prospect in coming years of expanding this therapeutic benefit and extending it to a greater range of clinical conditions. Nonetheless, the relatively low throughput and poor physiological relevance of assays that are currently available hamper clinical translation of BoNTs research. A major issue is in particular species-specificities of BoNTs activity; for example, the potency of BoNT/B is higher in mice than humans owing to a single-residue difference in the synaptotagmin 2 (SYT2) receptor resulting in a lower binding affinity of BoNT/B for human SYT2 [14]. In addition, the relatively high number of animals required for many current studies raises ethical issues [15–17].

In the past decade, the field has moved toward fully humanized in vitro models of the neuromuscular junction (NMJ) hoping to overcome both issue of throughput and relevance of rodent models. In this context, Yamanaka’s and Thomson’s [18–20] first description of human-induced pluripotent stem cells (hiPSCs) in 2007 was a major breakthrough. Human iPSCs are capable of unlimited self-renewal and can differentiate into any cell derivatives of the three germ layers. Because they are human by origin, hiPSC-derived NMJ has the potential to overcome both specie-specificities of BoNTs activity and to some extent poor throughput of assays in rodent. Two decades of research have provided differentiation protocols to generate the cell types involved in the NMJ including hiPSC-derived motor neurons and skeletal muscle cells [21, 22]. Formation of functional NMJ between hiPSC-derived motor neurons (hMNs) and rodent skeletal muscle [23], or primary human skeletal muscle [24–26], or even hiPSC-derived skeletal muscle [27, 28] has recently been described. In this context, several studies assessed BoNTs activity on hMNs using biochemical assays that confirmed cleavage of hMN’s synaptosomal-associated protein 25 KDa (SNAP25) by BoNTs [29–32]. Our group recently described a human motor endplate system where hMNs and immortalized human muscle cells were cocultured in the same well and showed that the system is sensitive to BoNT/A, evidencing reduced/blockade of muscle cells contractions exposed to BoNT [33]. Traditionally electrical stimulation (electrophysiology) is used to monitor and stimulate excitable cells but recent advances in optical-stimulation and calcium imaging methods open new avenues for the precise control and recording of individual compartment of NMJ modeled in vitro.

We built an in vitro model of humanized optogenetic engineering motor endplate using motor neurons derived from hiPSCs and immortalized human skeletal muscle cells. Our results confirmed that hMNs could innervate human muscle cells in vitro and generate a functional motor endplate model sensitive to BoNTs as identified by the measurement of contractions and intracellular calcium oscillations in skeletal muscle cells. In order to gain control on the pre-synaptic activity of our motor endplate model, we used optogenetics in hMNs. For this purpose, a red-shifted variant of channelrhodopsin, ReaChR, was expressed to generate blue-light sensitive hMNs and thus, gain optical control over their electrical activity as previously described [34–36]. We expressed GCaMP6f, a fluorescent fast genetically encoded calcium indicator, already known for its signal quality in myotubes [37], in our human muscular cells to monitor their activity in basal condition and upon drug or BoNTs exposure. Overall, we used our system to provide proof of concept of the coupling between hMN stimulation and muscle cell activity and to evaluate the sensitivity of humanized motor endplate to different BoNTs utilizing different mechanisms of action.

## Material and methods

### Human iPSCs culture and amplification

The WTSli020 hiPSC line from fibroblasts of dermis of a healthy female that was provided by EBiSC (European Bank for induced pluripotent Stem Cells) was cultured in feeder-free conditions using Vitronectin-coated culture vessels (VTN-N; Thermo Fisher Scientific, Waltham, MA, USA) and Essential 8 Flex medium (Gibco, Grand-Island, NY, USA) supplemented with Penicillin/Streptomycin (1:1000 PenStrep; Gibco). Another hiPSC line derived from a healthy female was also used as previously described [38]. Briefly, cells were thawed and manually expanded over five supplementary passages. For manual passaging, StemPro EZPassage tool (Thermo Fisher Scientific) was used. The automated cell culture system CompacT SelecT (Sartorius, Gottingen, Germany) was then used to generate a working cell bank using 0.25 mM EDTA (Thermo Fisher Scientific) in Phosphate-Buffered Saline (PBS; Gibco) without calcium or magnesium for cell passaging. Finally, cells were dispensed into cryovials using the automated system Fill-It (Sartorius) and frozen using CryoMed Controlled-Rate Freezer (Thermo Fisher Scientific). Quality controls (mycoplasma detection, pluripotency marker expression, genomic integrity) were performed before and after amplification.

### Flow cytometry

Cells were detached using TrypLE (Thermo Fisher Scientific) and resuspended at 100 000 cells per 50 µL in staining buffer containing PBS with 2 mM EDTA and Bovine Serum Albumin (0.5% BSA; Gibco). Antibodies, TRA1-81-AF647 (BioLegend, San Diego, CA, USA) and SSEA4-PE (Miltenyi Biotech, Bergisch Gladbach, Germany) were added at appropriate concentrations (according to the manufacturer’s instructions) and incubated for 30 min at 4 °C in the dark. Cells were analyzed on a MACSQuant flow cytometer (Miltenyi Biotec) with FlowJo software (Tree Star, San Carlos, CA, USA). A total of 20,000 events were recorded for each sample.

### Genomic integrity

Before passaging the cells, the cell culture supernatants of hiPSCs WTSli020-B cultures were collected and transferred directly into a safe-lock tube. The culture medium must have been in contact with the cells for at least 24 h. The supernatant samples containing cells were processed by Stemgenomics (St Eloi University Hospital Center, Montpellier, France) and analyzed using iCS-digital PCS test which detects by digital PCR more than 90% of recurrent genomic abnormalities in hPSCs supernatant (iCS-digital PSC 24-probes kit).

### Differentiation of hMNs progenitors from hiPSCs

Human iPSC embryoid body-based differentiation was performed as previously described by Maury et al. [21]. hiPSCs were dissociated with Accutase (Thermo Fisher Scientific) for 5 min at 37 °C and resuspended in basal medium which is a mix between DMEM-F12 Glutamax/Neurobasal (1:1 ratio; Gibco), N2 supplement/B27 no vitamin A supplement (1:2 ratio; Gibco), β-mercaptoethanol (0.1% β-ME; Gibco) and PenStrep (0.1%; Gibco), supplemented with small molecules including ascorbic acid (0.5 µM; Sigma-Aldrich), SB431542 (20 µM; TOCRIS-BioTechne, Minneapolis, MN, USA), LDN193189 (0.2 µM; Miltenyi Biotec), CHIR99021 (3 µM; Miltenyi Biotec) and Y-27632 (10 µM; STEMCELLS Technologies, Vancouver, Canada). Cells were seeded in suspension into T25 flask (Dutscher, Bernolsheim, France) to form embryoid bodies (EBs). During the entire culture process, small molecules were added at different timepoints including retinoic acid (0.1 µM RA; Sigma-Aldrich), Smoothened Agonist (0.5 µM SAG; STEMCELLS Technologies), Brain-Derived Neurotrophic Factor (10 ng/mL BDNF; PreproTech, Rocky Hill, NJ, USA) and γ-secretase inhibitor (10 µM DAPT; STEMCELLS Technologies). Then, EBs were dissociated at DIV 10 (days in vitro) and hMNs progenitors were generated. Cells were finally dispensed into cryovials and freezed using CryoMed Controlled-Rate Freezer (Thermo Fisher Scientific). The differentiation proceeded according to the schema presented among the figures.

### Human immortalized myoblasts culture

The human immortalized myoblasts cell line (AB1167c4, from fascia lata muscle of a healthy 20 years old male) was obtained from the MyoBank, Institute of Myology (Paris, France) [39]. Cells were seeded onto 96-well plate precoated with Collagen I (Thermo Fisher Scientific) at a concentration of 70,000 cells/cm^2^ in the myogenic induction medium composed of a mix between Medium 199/Dulbecco’s modified Eagle’s medium (DMEM) high glucose GlutaMAX (1:4 ratio; Gibco), Fetal Bovine Serum (20% FBS; Sigma-Aldrich, Saint-Louis, MO, USA), fetuin (25 µg/mL; Sigma-Aldrich), insulin (5 µg/mL; Gibco), Dexamethasone (0.2 µg/mL DXT; Sigma-Aldrich), Fibroblast Growth Factor (0.5 ng/mL FGF; STEMCELL Technologies), Epidermal Growth Factor (5 ng/mL EGF; STEMCELL Technologies) and gentamicin (0.1%; Gibco).

### Coculture of human immortalized myoblasts and hMNs

Human immortalized myoblasts were seeded onto 96-well plate and incubated over 24 h at 37 °C within a 5% CO_2_ environment in 96-well plate. The myogenic induction medium was replaced with coculture medium which was a mix between 1:3 of myogenic differentiation medium and 2:3 of hMNs growth medium. The myogenic differentiation medium was composed of DMEM high glucose GlutaMAX (Gibco), gentamicin (1%; Gibco) and insulin (10 µg/mL; Gibco). The hMNs growth medium was composed of a basal medium which is a mix between DMEM-F12 Glutamax/Neurobasal (1:1 ratio; Gibco), N2 supplement/B27 no vitamin A supplement (1:2 ratio; Gibco), β-mercaptoethanol (0.1% β-ME; Gibco) and Penicillin/Streptomycin (0.1% PenStrep; Gibco), supplemented with small molecules including ascorbic acid (0.5 µM; Sigma-Aldrich), retinoic acid (0.1 µM; Sigma-Aldrich), Smoothened Agonist (0.5 µM SAG; STEMCELLS Technologies), Brain-Derived Neurotrophic Factor (10 ng/mL BDNF; PreproTech, Rocky Hill, NJ, USA), Glial-Derived Neurotrophic Factor (10 ng/mL GDNF; PreproTech), γ-secretase inhibitor (10 nM DAPT; STEMCELLS Technologies). Y-27632 (10 µM; STEMCELLS Technologies) was used only for the thawing. hMNs progenitors generated in 10 days by following the protocol previously described in the section “Differentiation of hMNs progenitors from hiPSCs” were present at a concentration of 90 000 cells/cm^2^ and plated directly over the myoblasts and incubated at 37 °C with 5% CO_2_ for up to 15 days to mature and differentiate. Half of coculture medium was changed every 5 days.

### Immunostaining

Cells were fixed with 4% paraformaldehyde for 15 min and further permeabilized and blocked with Triton X-100 (0.1%; Sigma-Aldrich) and BSA (2%; Gibco) in PBS (Gibco) for 30 min. Primary antibodies were then added and were incubated at 4 °C overnight in PBS/BSA/Triton solution. Human iPSCs were labelled with Nanog (1:1000; Abcam, Cambridge, UK), Oct4 (1:400; Thermo Fisher Scientific), Sox2 (1:200; Thermo Fisher Scientific) and TRA1-60 (1:500; Abcam). Neurons were labelled with Tuj1 (1:1000; BioLegend), Islet1 (1:200; Neuromics, Edina, MN, USA), Synaptophysin (1:100 SYN; Abcam), Neurofilament H Non-Phosphorylated (1:200 SMI32; Abcam) and SNAP25 (1:1000; Sigma-Aldrich) antibodies. Myotubes were labelled with Rhodamine-Phalloidin (1:200; Invitrogen, Carlsbad, CA, USA), Myosin-Alexa Fluor 488 (1:500 MF20; Invitrogen) and Sarcomeric Alpha Actinin (1:200 SAA; Sigma-Aldrich) antibodies. The clusters of acetylcholine receptors were labelled with AChR antibody (1:100; DSHB, Iowa City, IA, USA). Appropriate Alexa Fluor 488/594/647-conjugated secondary antibodies (1:1000; Invitrogen) were used with 4′,6-diamidino-2-phenylindole (DAPI) nuclear counterstain (1:1000; Invitrogen) for 2 h at room temperature. Staining was visualized and imaged on an Evolve EMCCD camera (Zeiss, Oberkocen, Germany) coupled to a Spinning Disk system (Nipkow, CSU-X1M 5000; Zeiss) with Metamorph (Molecular Devices, San José, CA, USA). Images were processed with Photoshop 2020 (Adobe, Mountain View, CA, USA) and ImageJ version 1.52 (NIH, Bethesda, MD, USA). To quantify Islet1^+^ hMNs, cells were counted with the aid of the Cell Counter plugin for ImageJ. To quantify myotubes fusion index, the ratio of the nuclei number in MF20^+^ myotubes with ≥ 3 nuclei versus the total number of nuclei was calculated with the aid of an ImageJ plug-in determining the number of nuclei at which another staining of interest is colocalized.

### RNA extraction and quantitative reverse transcriptase chain reaction (RT-qPCR)

hMNs were plated onto 24-well plate. Total RNA from two wells was isolated with the PicoPure RNA Isolation Kit (Applied Biosystems, Foster City, CA, USA) according to the manufacturer’s instructions. Human total adult Spinal Cord RNA (BioChain Institute Inc., Newark, CA, USA) was used as positive control. cDNA was generated from 0.5 μg of RNA with High Capacity cDNA Reverse Transcription kit (Applied Biosystems). Quantitative real time-polymerase chain reactions (QRT-PCRs) were performed with the TaqMan Universal PCR Master Mix (Applied Biosystems) and the following TaqMan Human Gene Expression Assays: ISLET1 (Hs00158126_m1); ACHE (Hs01085739_g1); CHAT (Hs00252848_m1); SYP (Hs00300531_m1); SNAP25 (Hs00938962_m1); VAMP1 (Hs01042310_m1); VAMP2 (Hs00360269_m1); VAMP3 (Hs00922166_m1); SV2A (Hs01059458_m1); SV2B (Hs00208178_m1); SV2C (Hs00392676_m1); SYT1 (Hs00194572_m1); SYT2 (Hs00980604_m1); and GAPDH (Hs03929097_g1). Quantification was performed at a threshold detection line (Ct value). The Ct of each target gene was normalized to GAPDH housekeeping gene.

### Botulinum neurotoxins treatment

Cells were exposed to several doses of recombinant BoNT/A (rBoNT/A) produced from *Escherichia Coli* as previously described [40] or modified BoNT (mBoNT). Both toxins were engineered and purified to more than 90% by IPSEN (Milton Park, UK). Each dose of BoNTs was tested in triplicate and a negative control (toxin-free medium) was always included. For Western blotting experiment, after 24 h the medium containing the toxin was removed, cells were washed with PBS and lysed.

### SNAP25 cleavage assay by Western blot

Cells protein lysis and Western blot were performed as previously described [33]. Bands were visualized on an ImageQuant LAS 4000 (GE Healthcare Life Sciences, Marlborough, MA, USA) and processed with Image Studio Lite (LI-COR Biosciences, Lincoln, NE, USA). For analysis, the half maximal effective concentration (EC_50_) was calculated with GraphPad Prism version 8.3 (GraphPad Software).

### Muscle cells contractions and Ca^2+^ transient measurements

Cocultures at DIV 15 were placed in 96-well plate in a live imaging system with an Evolve EMCCD camera coupled to a Spinning Disk system (Zeiss), and timelapse imaging (1 min film with time interval of 300 ms for each recording) was performed under physiological conditions (37 °C and 5% CO_2_). Recording was done in a same myofiber for each condition. Recording was done in basal condition before and after any drug addition, i.e., 5 µM tetrodotoxin (TTX; Tocris Bioscience, Bristol, UK), 150 µM tubocurarine (Sigma-Aldrich), rBoNT/A (IPSEN). For Ca^2+^, measurements cells were loaded for 15 min with 2 µM Cal520 (Abcam, Cambridge, UK), which is a Ca^2+^ indicator, dissolved in loading buffer, then washed 3 times with recording buffer. The loading buffer was composed of a mix between 10X Hanks' Balanced Salt Solution (HBSS) calcium magnesium/distilled water (1:8 ratio; Gibco), 1 M hepes (20 mM; Gibco) and sodium hydroxide (2 mM NaOH; Sigma-Aldrich). The recording buffer was composed of a mix between 10X HBSS/distilled water (1:8 ratio; Gibco), 1 M hepes (20 mM; Gibco), sodium hydroxide (2 mM NaOH; Sigma-Aldrich) and calcium chloride (2 mM CaCl_2_; Sigma-Aldrich). For each recording (contractions and Ca^2+^), we used three myofibers/well and three wells/condition. Myotube contraction analyses were performed with an open-source software tool MUSCLEMOTION following the instructions from the software [41]. This software quantifies movement by subtracting the summed, absolute changes in pixel intensity between a reference frame and the frame of interest. Ca^2+^ oscillation analyses were performed with the semi-automated open-source Ca^2+^ imaging analyzer CALIMA to detect Ca^2+^ activity in myotubes by following the instructions from the software [42]

### Adeno-associated virus transduction

The photoactivated domain ReaChR was obtained from Addgene (Watertown, MA, USA) under human Synapsin 1 (hSyn1) promoter (Plasmid #50954) as well as the plasmid containing the calcium sensor GCaMP6f under Human cytomegalovirus (CMV) promoter (Plasmid #40755). Both plasmids were packaged into Adeno-associated virus serotype 2 (AAV2) with a functional titer of 10^13^ GC/mL by Vigene Biosciences (Rockville, MD, USA). To define the best MOI for ReaChR and GCaMP6f transduction, muscle cells and hMNs were exposed to viral doses ranging in MOI of control vectors AAV2-CMV-GFP (Applied Biological Materials, Richmond, Canada) for muscle cells and AAV2-hSyn1-GFP (Vigene Biosciences) for hMNs. To quantified GFP^+^ control signal in hMNs and in muscle cells, colocalization between GFP^+^/Phalloidin^+^ muscle cells and GFP^+^/Islet1^+^ hMNs was counted using the ImageXpress Micro Confocal High-Content Imaging System (Molecular Devices, San José, CA, USA). After selecting the appropriate MOI, muscle cells were transduced at DIV 1 post-seeding with AAV2-CMV-GCaMP6f-WPRE-SV40pA construct with 5000 MOI, and hMNs were transduced with AAV2-hSyn1-ReaChR-citrine construct at DIV 8 post-seeding hMNs over muscle cells with 10 000 MOI.

### Optogenetic stimulation procedure and Ca^2+^ measurements

Optogenetic imaging experiments were performed in specific recording buffer where detailed in the section “Muscle cells contractions and Ca^2+^ transient measurements” above. Red light stimulation of cells was triggered with a Fiber-Coupled LED 590 nm (Thorlabs, Newton, NJ, USA) connected to a DC4100 4-Channel LED Driver (Thorlabs) in live imaging system with a Spinning Disk (Zeiss) under physiological conditions. ReaChR^+^ hMNs were activated at 20 mW/cm^2^ intensity with the optical fiber by 20 pulses of light, each pulse having a duration of 20 ms. Recordings were acquired with Metamorph (Molecular Devices). The addition of drugs was performed for optogenetics assay: 150 µM tubocurarine (Sigma-Aldrich), 10–50 µM range of glutamate (Sigma-Aldrich), BoNTs (IPSEN). For BoNTs assay, recordings were performed before addition and 4 h/8 h after exposure. For all recording, we used three myofibers/well and three wells/condition. Ca^2+^ oscillation analysis was performed using ImageJ version 1.52 (NIH) with Region of Interest (ROI) manager and using CALIMA software [42]. Fluorescence changes in GCaMP6f^+^ myotubes were expressed as the ratio F590/F0 normalized to basal values (ΔF/F0). Subsequently, the transmission of emission light intensity was quantified with a thermophile UNO laser power meter (Gentec Electro-Optics, Québec, Canada).

### Statistics

All statistical analysis was performed using GraphPad Prism version 8.3 (GraphPad Software). One-way ANOVA with Sidak’s post hoc tests was used to determine statistical significance (*^,#^*p* < 0.05; **^,##^*p* < 0.01; ***^,###^*p* < 0.001; ****^,####^*p* < 0.0001; ns, not significant).

## Results

### Production of human motor neurons from iPSCs to study BoNT activity

In order to secure large quantity of cells for this study, we produced and quality controlled undifferentiated hiPSCs using a CompacT SelecT automate as previously described [43] (Additional file 1: Fig. S1a, b, c). In the same line, hMNs progenitors were produced (Additional file 2: Fig. S2a) and terminally differentiated into post-mitotic motor neurons as previously described [21]. To control the cellular identity of our neuronal population, we assessed the expression of key markers by RT-qPCR and immunofluorescence after 14 days in vitro (DIV 14). RT-qPCR analysis revealed expression levels of neuronal (MAP2), hMNs (Islet1), cholinergic (AChE, ChAT) and synaptic (SYP) markers at least tenfold and up to 100-fold higher than expression in human total adult spinal cord control (Additional file 2: Fig. S2b). Immunostaining confirmed the expression of Islet1 and Tuj1 markers in hMNs (Additional file 2: Fig. S2c). This confirmed the robustness of the protocol to obtain highly enriched hMNs with the WTSli020-B hiPSC line. Next, to assess the relevance of such hMNs for BoNTs studies, we measured the expression level of BoNT SNARE substrates and BoNT receptors. RT-qPCR data showed that hMN expression level of BoNT substrates: synaptosomal protein (SNAP25), vesicle-associated membrane protein (VAMP1/2/3), and BoNT receptors: synaptic vesicle 2 isoforms A, B, and C (SV2A/B/C), and synaptotagmin isoforms 1, and 2 (SYT1/2), was at least fivefold and up to 100-fold higher that our reference spinal cord sample (Additional file 2: Fig. S2b). Overall, these results suggested that hMNs were suitable for BoNTs studies.

### Characterization of the muscle-nerve coculture and evidence of motor endplate formation

**Fig. 1:**
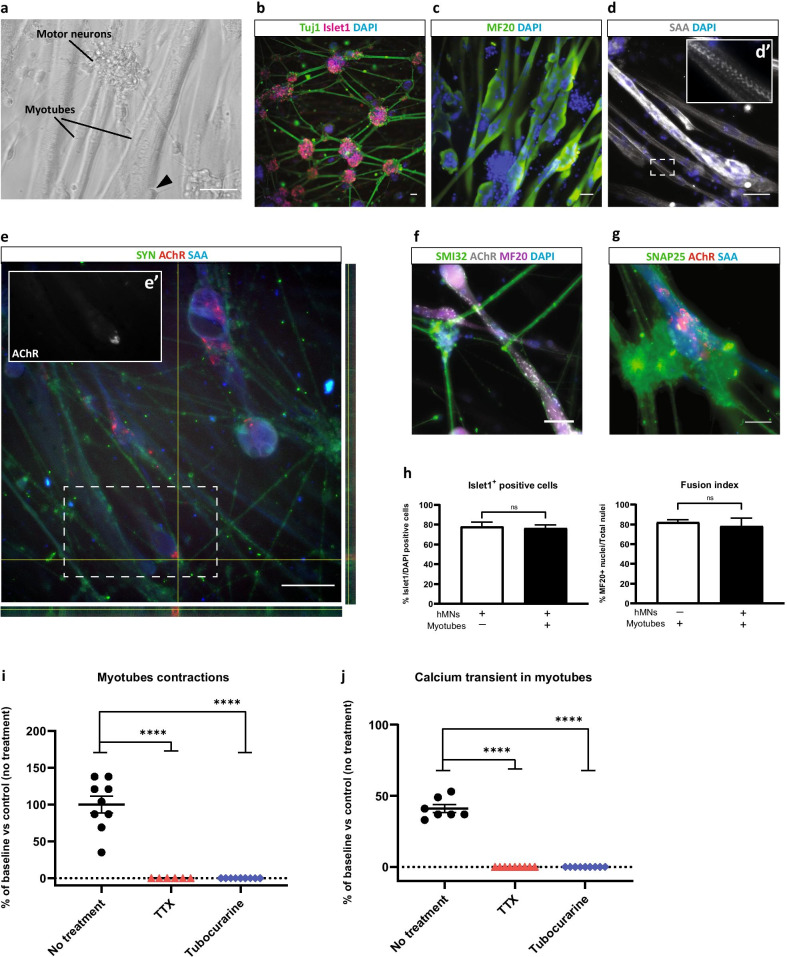
Generation of an in vitro functional muscle-nerve coculture system at DIV 15. **a** Representative images of the muscle-nerve coculture where hMNs neurites made contacts with myotubes (black triangle in the phase contrast image). **b** hMNs express Islet and Tuj1. **c**, **d/d’** Myotubes express MF20 and SAA. **e/e’**–**g** The coculture system expresses SYN (right and lower panels represent cross sections of myotubes in the orthogonal view), SMI32, SNAP25 and presents AChR clusters. **a**–**g** Scale bars: 100 µm. **h** Quantification of Islet1^+^ hMNs and quantification of fusion index in myotubes. Data are represented as mean ± SD (*N* = 3 independent experiments, each performed in triplicate *n* = 3). Mann–Whitney test (ns., not significant). **i** Disruption of myotubes contractions after treatment with 5 µM TTX and 150 µM tubocurarine compared to control condition with no treatment. Each drug was added at DIV 15, the recording of myotubes contractions was performed before treatment (baseline) and 30 min after treatment. **j** Disruption of Ca^2+^ oscillations in myotubes after treatment with 5 µM TTX and 150 µM tubocurarine compared to control condition with no treatment. For Ca^2+^, measurement cells were stained with 2 µM Cal520 dye the day of the recording. Each drug was added at DIV 15, the recording of Ca^2+^ oscillations was performed before treatment (baseline) and 30 min after treatment. **i**, **j** Data are represented as mean ± SEM (*N* = 3 independent experiments, each performed in triplicate *n* = 3). ANOVA with Sidak’s post hoc tests (****p < 0.0001)

We next sought to evaluate the capacity of hMNs to functionally interact with skeletal muscle cells, and DIV 10 hMNs progenitors were seeded directly over immortalized human myotubes (AB1167c4 line from the MyoBank, Institute of Myology, Paris) (Additional file 3: Fig. S3). The differentiation of both hMNs and myotubes was observed after 15 days of coculture and showed that hMNs developed a typical morphology of mature neurons with a tendency to form cell body clusters and with axons terminating on large myotubes (Fig. 1a).

To confirm the identity and maturity of each type of cells in our muscle-nerve in vitro model, we immunostained the coculture with antibodies against neuron-specific class III tubulin (Tuj1) and Islet1 a transcription factor expressed in post-mitotic hMNs to identify the neuronal component of the coculture. Myogenic markers such as Myosin Heavy Chain (MF20) and sarcomeric alpha actinin (SAA) were also analyzed to identify multi-nucleated striated myotubes. Islet1^+^ nuclei tended to be grouped into clusters from which bundles of Tuj1^+^ neurites extended (Fig. 1b). MF20-positive soma containing multiple DAPI-stained nuclei exhibited striated band patterns stained with SAA suggesting the formation of the contractile system for muscle cells (Fig. 1c–e). We next analyzed the presence of neuromuscular synapses using antibodies against markers of the presynaptic component (Neurofilament SMI32, synaptophysin/SYN and synaptosomal protein SNAP25) and against the acetylcholine receptor (AChR) in the post-synaptic component of the NMJ (Fig. 1e–g). Multiple clusters of SYN^+^ staining juxtaposed with AChR clusters along myotubes suggesting the formation of motor endplate (Fig. 1e). Validating these observations, we detected puncta of synaptosomal protein SNAP25 in hMNs at the close proximity of AChR clusters (Fig. 1g). To assess whether coculture of hMNs with muscle cells had an impact on either cell maturation, we quantified the percentage of Islet1^+^ hMNs in mono-culture and in coculture. We evidenced neither enhanced or decreased percentage of viable Islet1^+^ hMNs in coculture compared to hMNs mono-culture (76.45% and 78.06% on average, respectively, *p* = 0.28) (Fig. 1h, left). Likewise, the fusion index of myogenic cells was found to be similar independently of the presence of hMNs (Fig. 1h, right).

### Human muscle-nerve coculture form functional motor endplate

At the functional level, myotube contractions were observed after 15 days of cocultures, whereas no contractions were detected in myotubes in mono-culture suggesting that hMNs input was necessary for such stimulation of muscle cells (Additional file 8: Video S1 and Additional file 9: Video S2). We next challenged the muscle-nerve system with TTX (5 µM), a voltage-dependent sodium channels blocker, or tubocurarine (150 µM), an AChRs antagonist, to assess whether muscle contractions were mediated by action potential-dependent synaptic activity and ACh neurotransmission. Both TTX and tubocurarine blocked myotube contractions in muscle-nerve coculture (*p* < 0.0001) (Fig. 1i, Additional file 10: Video S3 and Additional file 11: Video S4). Because variation of pixel intensities used to quantify myotube contractions tented to vary from preparation to preparation and over time and thus make this read-out difficult to exploit, we tested the monitoring of calcium level in myotubes as an alternative to myotubes contractions. The fluorogenic Ca^2+^-sensitive indicator, Cal520, was therefor used to quantify Ca^2+^ transient in skeletal muscle cells in the presence or absence of hMNs. Modulation of fluorescence intensity was observed in skeletal muscle cells only in the presence of hMNs (Fig. 1j). A pharmacological approach was used to further confirm the specific and functional connectivity between hMNs and skeletal muscle cells. Then, treatment with TTX and tubocurarine completely abolished Ca^2+^ transient in myotubes in the presence of hMNs (*p* < 0.0001) (Fig. 1j and Additional file 12: Video S5). Altogether, our results suggest the presence of a functional motor endplate in these humanized coculture systems.

### *Effect of BoNTs on *in vitro* human motor endplate*

**Fig. 2:**
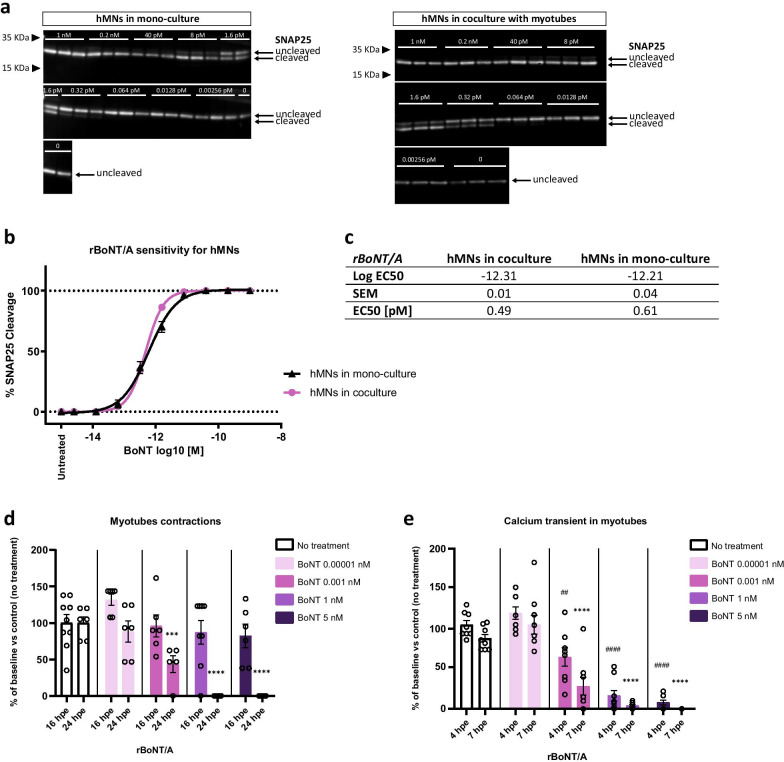
Effect of botulinum neurotoxin on muscle-nerve coculture function. **a** Detection by Western blot of cleaved-SNAP25 from hMNs mono-culture (left) or coculture (right) treated with serial doses of rBoNT/A, compared to toxin-free control dose (untreated) using an antibody recognizing cleaved and uncleaved form of SNAP25 protein. **b** EC_50_ curve for hMNs in mono-culture or in coculture. DIV 15 hMNs were exposed to rBoNT/A for 24 h before cell lysates were harvested, followed by Western blot to quantify SNAP25 cleavage. **c** EC_50_ for hMNs in coculture is 0.49 pM (10^–12.31^) and EC_50_ for hMNs in mono-culture is 0.61 pM (10^–12.21^). Data are represented as mean ± SEM (*N* = 3 independent experiments, each performed in triplicate *n* = 3). **d** Effect of rBoNT/A on myotubes contractions 16 hpe (hour post-exposure) and 24 hpe. Coculture was treated at DIV 15 with different doses of rBoNT/A (5 nM, 1 nM, 0.001 nM, 0.00001 nM), and recordings of myotubes contractions were performed before treatment (baseline), 16 h and 24 h after treatment. Data are represented as mean ± SEM (*N* = 3 independent experiments, each performed in triplicate *n* = 3). ANOVA with Sidak’s post hoc tests (***p < 0.001; ****p < 0.0001). Significant statistics only 24 hpe, each dose was compared to untreated condition. **e** Effect of rBoNT/A on myotubes Ca^2+^ oscillations 4 hpe and 7 hpe. Cells were stained with 2 µM Cal520 dye the day of the recording. Coculture was treated at DIV 15 with different doses of rBoNT/A (5 nM, 1 nM, 0.001 nM, 0.00001 nM), and recordings of Ca^2+^ oscillations were performed before treatment (baseline), 4 h and 7 h after treatment. **d-e** Data are represented as mean ± SEM (*N* = 3 independent experiments, each performed in triplicate *n* = 3). ANOVA with Sidak’s post hoc tests (^##^*p* < 0.01; ^####,^****p < 0.0001). Significant statistics 4 hpe (represented by ^#^) and 7 hpe (represented by *), each dose was compared to untreated condition

We next sought to evaluate the potential of this humanized coculture system for testing BoNTs. As the mechanism of BoNT action involves the hydrolysis of the proteins of the SNARE complex [13, 44], the cleavage activity of rBoNT/A, a recombinant BoNT of serotype A, on SNAP25 was evaluated by Western blot analysis after treatment of hMNs alone or cocultured with skeletal muscle cells. This analysis revealed a dose-dependent activity of the toxin independently of the cellular system. In addition, similar EC_50_ was found for hMNs alone or cocultured with skeletal muscle cells (0.61 pM and 0.49 pM, respectively) (Fig. 2a–c). These biochemical analysis confirmed that the humanized in vitro model of human motor endplate was as sensitive to BoNT/A as hMNs alone and that the potency of the toxin on our system was similar to that previously observed with the same BoNT with another source of hMNs [31, 33].

We assessed the ability of four specific doses of rBoNT/A, consistent with the range of toxin doses previously used for the first Western blotting, to affect myotube contractions and Ca^2+^ transient over time using live video microscopy. We observed an interruption in frequency of myotube contractions after 24 h of rBoNT/A treatment with the two highest doses of 1 nM and 5 nM (for both doses: *p* < 0.0001) (Fig. 2d). Treatment with lower doses of rBoNT/A (0.001 nM and 0.00001 nM) only partially decreased the myotube contractions (Fig. 2d). After 16 h of exposure with rBoNT/A, no decrease in myotube contractions was observed (Fig. 2d). Twofold decrease in frequency or complete blockade of Ca^2+^ transient in myotubes was similarly observed but at earlier time post-exposure (4 h and 7 h) after rBoNT/A treatment with the lowest dose of 0.001 nM (*p*_4hpe_ < 0.01 and *p*_7hpe_ < 0.0001) and the two highest dose of 5 nM and 1 nM (for both doses: *p*_4hpe_ < 0.0001 and *p*_7hpe_ < 0.0001), respectively (Fig. 2e). Altogether, these results indicated the relevance of recording BoNT activities by using hiPSC-based cellular model of NMJ. In complement to biochemical analysis, this cellular system also offers the possibility to monitor longitudinally functional read-outs such as muscle contractions and Ca^2+^ transient. Accordingly, we validated that the EC_50_ for SNAP25 cleavage was similar to the concentration necessary to decrease by half the frequency of muscle contraction or Ca^2+^ transient (Additional file 4: Fig. S4a, b).

### ***Light activation of ReaChR in hMNs induce Ca***^***2***+^***changes in myotubes***

**Fig. 3:**
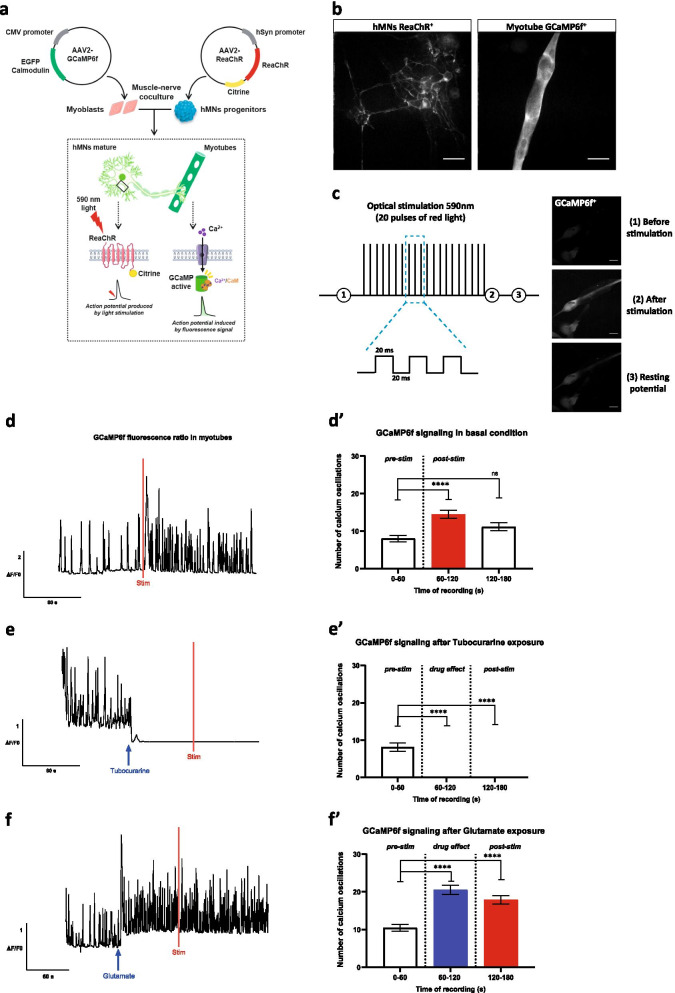
Effect of the optogenetic stimulation of hMNs on the myotubes Ca^2+^ dynamic. **a** ReaChR was transduced into hMNs progenitors to enable ion channel activation by light allowing for control of neuronal activity by red light stimulation (590 nm). Optogenetic activation was confirmed by reading the Ca^2+^ response generated in myoblasts transduced with GCaMP6f by quantifying fluorescent intensity. **b** AAV2-hSyn-ReaChR-citrine expression in hMNs for optogenetic control and AAV2-CMV-GCaMP6f-WPRE-SV40pA expression in myotubes. Scale bars: 50 µm.** c** Representative schema of the optical stimulation protocol at 590 nm: 20 red light pulses, each pulse was 20 ms long. Optogenetics activation was confirmed by GCaMP6f in myotubes by quantifying fluorescent intensity. Scale bars: 50 µm. **d-d’** Representative traces of normalized GCaMP6f fluorescence before and after red light stimulation (red bar) and quantification of Ca^2+^ oscillations. Data are represented as mean ± SEM (*N* = 3 independent experiments, 3 myofibers/well, 3 wells/condition). ANOVA with Sidak’s post hoc tests (****p < 0.0001; ns, not significant), with the recording times 60–120 and 120–180 compared to the time 0–60. **e-e’** Effect of the addition of 150 µM tubocurarine before and after red light stimulation (red bar) and quantification of Ca^2+^ oscillations. Data are represented as mean ± SEM (*N* = 3 independent experiments, 3 myofibers/well, 3 wells/condition). ANOVA with Sidak’s post hoc tests (****p < 0.0001), with the recording times 60–120 and 120–180 compared to the time 0–60. **f**–**f’** Effect of the addition of 10–50 µM range of glutamate before and after red light stimulation (red bar) and quantification of Ca^2+^ oscillations. Data are represented as mean ± SEM (*N* = 3 independent experiments, 3 myofibers/well, 3 wells/condition). ANOVA with Sidak’s post hoc tests (****p < 0.0001), with the recording times 60–120 and 120–180 compared to the time 0–60

With the aim of allowing longitudinal monitoring of our model of humanized motor endplate while avoiding the toxicity of long-term exposure to Ca^2+^ dyes such as Cal520 [45], we used genetically encoded Ca^2+^ indicator GCaMP6f. We transduced human myoblast with the GCaMP6f in order to gain access to an easy way to monitor longitudinally Ca^2+^ transients only in muscle cells (Fig. 3a). To obtain a constitutive expression in myotubes, the expression of Ca^2+^ sensor was placed under the CMV promoter. Using an AAV2-CMV-GFP reporter virus, we first determined that a MOI of 5000 was necessary to achieve optimal skeletal muscle cell transduction (Additional file 5: Fig. S5a–c). In order to gain control over the neuronal activity of our functional motor endplate, we chose the use of ReaChR optogene whose expression was placed under the human synapsin promoter to ensure transgene expression restricted to only neurons (Fig. 3a). Similarly, using an AAV2-hSyn1-GFP reporter virus we determined that a MOI of 10 000 was required to efficiently transduce hMNs (Additional file 5: Fig. S5a, d, e). We next validated that indeed ReaChR expression was restricted to hMNs, whereas expression of GCaMP6f was detected in the cytoplasm of polynuclei skeletal muscle cells (Fig. 3b).

In order to determine the adequate parameters to achieve optimal stimulation, the light power intensity depending on the current applied by the laser at 590 nm was measured. We consequently chose to work with the maximum capacity of the laser corresponding to a light power of 20 mW/cm^2^ applied on the cells in our optogenetics platform (Additional file 5: Fig. S5f, g). We next determined that the optimized stimulation of ReaChR^+^ hMNs was induced by 20 red light pulses of 20 ms each (Fig. 3c). Because hMNs are spontaneously active, we observed spontaneous Ca^2+^ oscillations in skeletal muscle cells before light stimulation. Then, light stimulation was found to induce increased Ca^2+^ oscillations frequency with a higher Ca^2+^ oscillation following the pulses of light (Fig. 3d and Additional file 13: Video S6). The quantification of Ca^2+^ oscillations clearly showed an increase within 60 s post-stimulation (p_60-120_ < 0.0001) and a recovery the following 60 s (Fig. 3d’).

To fully demonstrate the potential of the optogenetic approach to analyze the functional communication between hMNs and human skeletal muscle cells, we tested several drugs known to inhibit or activate the activity of the muscle-nerve system. As expected tubocurarine prevented light-induced increase in the frequency of Ca^2+^ transients in myotubes (Fig. 3e and Additional file 14: Video S7), that was confirmed with the quantification of Ca^2+^ oscillations showing the inhibiting effect (p_60-120_ < 0.0001 and p_120-180_ < 0.0001) (Fig. 3e’). In contrast, glutamate stimulation triggered an increase in Ca^2+^ transients in myotubes that remained unchanged after light stimulation (p_60-120_ < 0.0001 and p_120-180_ < 0.0001) (Fig. 3f, f’). Altogether, the results demonstrated that skeletal muscle cells’ activity could be induced by light-activated hMNs. Our results also demonstrated that humanized in vitro cell model of NMJ are sensitive to pre-synaptic stimulation (glutamate) or post-synaptic inhibitor (tubocurarine). Thus, humanized in vitro cell model of NMJ combined with optogenetics tool can further be compatible with analysis of functional connectivity between ReaChR^+^ hMNs and GCaMP6f^+^ myotubes.

### *Impact of BoNTs exposure on optogenetic controlled human motor endplate *in vitro

**Fig. 4:**
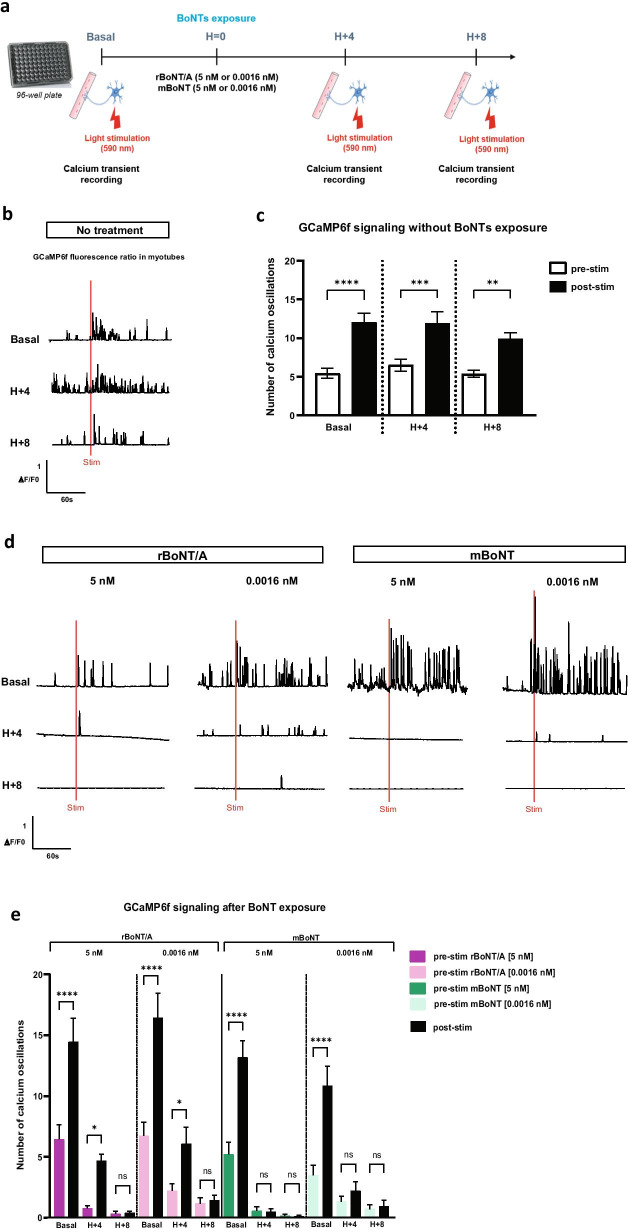
BoNTs effects on optogenetic controlled muscle-nerve system. **a** Representative schema of the optogenetic procedure before (baseline/before treatment) and 4 h and 8 h after exposure of BoNTs (H + 4 and H + 8). The addition of both BoNTs was notified by H = 0. Cells were exposed to two doses (5 nM or 0.0016 nM) of rBoNT/A or mBoNT. **b**, **c** Representative traces of normalized GCaMP6f fluorescence in untreated condition before (pre-stim) and after (post-stim) red light stimulation (red bar) over the time and quantification of Ca^2+^ oscillations. Data are represented as mean ± SEM (*N* = 2 independent experiments, 3 myofibers/well, 3 wells/condition). ANOVA with Sidak’s post hoc tests (****p < 0.0001; ***p < 0.001; **p < 0.01), each pre-stim recordings were compared to post-stim recordings. **d**, **e** Representative traces of normalized GCaMP6f fluorescence at 5 nM rBoNT/A, 0.0016 nM rBoNT/A, 5 nM mBoNT and 0.0016 nM mBoNT treatments before and after red light stimulation (red bar) over the time and quantification of Ca^2+^ oscillations. Data are represented as mean ± SEM (*N* = 2 independent experiments, 3 myofibers/well, 3 wells/condition). ANOVA with Sidak’s post hoc tests (****p < 0.0001; *p < 0.05; ns, not significant), each pre-stim recordings were compared to post-stim recordings

The adaptability of the light-sensitive humanized in vitro system to decipher the effects of BoNTs was next evaluated. We compared the effect of two different toxins, rBoNT/A and modified BoNT (mBoNT), to assess whether our system could evidence different activities or signs of different mechanism of action. We chose to record the Ca^2+^ activity in GCaMP6f^+^ myotubes in response to optical stimulation of ReaChR^+^ hMNs before and after exposure to toxins for 4 h and 8 h, compared to control condition with no treatment (Fig. 4a). We confirmed that the light stimulation at 590 nm increased Ca^2+^ oscillations in skeletal muscle cells over time when cells were not treated (*p*_basal_ < 0.0001; *p*_H+4_ = 0.0004 and *p*_H+8_ = 0.0038) (Fig. 4b, c). In contrast, a progressive reduction in Ca^2+^ oscillations was observed in skeletal muscle cells independently of the doses of rBoNT/A and independently of optical stimulation. Almost complete inhibition was observed after 8 h post-exposure with the highest dose at 5 nM (Fig. 4d-e). A similar effect was observed with the mBoNT, but in this case the highest dose of 5 nM resulted in an almost total disruption of the Ca^2+^ oscillations as soon as 4 h post-exposure, whereas this effect occurred 8 h post-exposure with the lower dose of 0.0016 nM (Fig. 4d-e). The effect observed with mBoNT showed a higher potency compared to rBoNT/A. Indeed, optical stimulation 4 h after rBoNT/A exposure slightly increased Ca^2+^ oscillations in muscle cells (for 5 nM *p*_H+4_ = 0.0427 and for 0.0016 nM *p*_H+4_ = 0.0486) which is not the case after exposure to mBoNT. This result was concordant with the biochemical analysis of SNAP25 cleavage measured by Western blotting in hMNs after 24 h of exposure with mBoNT in which an EC_50_ of 0.01 pM as calculated (Additional file 6: Fig. S6a, b). In order to evaluate the reliability of our approach, we next sought to extend our results by using an independent hiPSC line. We thus confirmed that under basal conditions, light stimulation increased Ca^2+^ oscillations in skeletal muscle cells, whereas a reduction in Ca^2+^ oscillations was similarly observed after treatment with the reference toxin rBoNT/A (Additional file 7: Fig. S7).

Altogether, these data confirmed that the capacity of light-sensitive humanized in vitro NMJ model to determine the effect of BoNTs as soon as 4 h after treatment, but also for comparative analysis of different BoNTs. This comparison between BoNTs highlighted the possibility to study difference in onset of action and duration of action, which could represent a considerable advantage over the routine Western blot test for BoNTs potency measurement. Indeed, light-sensitive humanized in vitro NMJ could represent a faster and more physiological system for the field of BoNTs.

## Discussion

The main finding of this study is the description of a novel in vitro humanized optogenetic engineering motor endplate model which is based on the differentiation of hiPSC-derived motor neurons and immortalized human skeletal muscle cells. In this system, hMNs established functional synaptic contact with human myotubes that are sensitive to pharmacological challenge of hMNs synaptic activity and ACh neurotransmission. To gain a better control and ease to monitor neuromuscular activity, we genetically engineered our motor endplate model with neuronal optogene (ReaChR) and muscular-calcium sensor (GCaMP6f).

The increased incidence and prevalence of inherited neuromuscular diseases have led to the development of pertinent pathological models for deciphering the pathophysiology underlying these diseases and developing effective treatments [46]. Although animal models have been crucial in this field, certain limitations still exist in the form of ethical issues involving animal welfare or the capacity of animal models to fully reproduce some human pathological phenotypes. To overcome these hurdles, different models and approaches have been developed to recreate in vitro the NMJs. More specifically, the recent advances in human pluripotent stem cell biology have fueled the prospect of generating the main components of the NMJ and consequently humanized NMJ in vitro [47]. In the last five years, different studies have already described the possibility of using human pluripotent stem cells to generate optogenetic human motor endplate models for disease modeling applications [48–51]. Concordant with these different reports, our results also validate the potential of human pluripotent stem cells combined with optogenetic technology to recreate a functional humanized NMJ system. Whereas most of these studies described 3D culture models, our study, however, strengthens the potential of using 2D system. In addition, identifying muscle fibers has been shown to be challenging [26]. In this study, we validate the possibility of combining fluorescently labeled skeletal muscle cells (e.g., GCaMP6f^+^) with light sensitive hMNs to improve the detection of innervated skeletal muscle cells in cocultures and avoid toxicity that may be associated with the use of synthetic calcium dye [36, 45, 48, 49, 52, 53].

Most of the attempts to develop humanized in vitro motor endplates have focused so far on their potential use for disease modeling. Thus, different studies have already demonstrated the potential use of these in vitro models for myasthenia gravis, amyotrophic lateral sclerosis or spinal muscular atrophy [49–51, 54]. Our results also extend the potential of humanized in vitro models of motor endplate for pharmacological applications such as the field of BoNTs. As a first step, we validated that human-induced pluripotent stem cells-derived motor neurons express all the different actors known to be involved in BoNTs intoxication process (i.e., SNARE proteins and BoNT protein receptors). Then, by evaluating one the best characterized BoNT serotypes (BoNT/A), our results indicate that humanized in vitro motor endplate harbor a level of sensitivity to recombinant BoNT/A (rBoNT/A engineered by IPSEN) similar that what is observed with hMNs alone, also with commercial hMNs used for another study by our team [33]. Our results also suggest that hMNs used in this study harbor a higher sensitivity compared to others hMNs models recently described [31, 32]. These differences might arise from the origin of BoNT used, protocols for treatment, hMNs differentiation and culture, or in read-out sensitivity [31, 32]. Strikingly, our results did not reveal differences in sensitivity to toxins between hMNs alone or cocultured with skeletal muscle cells. It remains possible that addition of Schwann cells, another important cellular component of NMJ, to the co-culture might help to better mimic the native structure of synapses and could increase the maturity of the system [55–57]. To further validate that the presynaptic activation of hMNs caused changes in muscle activity, we evaluated the functional effect of rBoNT/A in the coculture. The data revealed that rBoNT/A reduced the activity of the cocultured muscle cells and confirmed the use of calcium transient read-out as a relevant measurement for the activity of BoNTs only few hours after treatment.

Optogenetics technology represents an opportunity for drug screening and disease modeling in the field of BoNT studies. The use of this technology could be applied to study the onset and duration of action of different BoNTs, and to model different neuromuscular disorders such as dyskinesia and spasticity, with distinct activity profile NMJs. In this context, we successful developed a novel metric for monitoring the effect of two BoNTs on in vitro functional human motor endplate. This is the first time that two toxins are compared on a controlled in vitro human motor endplate model. Our investigations revealed that both toxins, rBoNT/A and mBoNT (engineered by IPSEN), thus acted efficiently on hMNs in a dose-dependent manner. Interestingly, we noted a difference in the sensitivity of the coculture system between these toxins. The inhibition of neurotransmission was faster after mBoNT exposure than rBoNT/A exposure. This could be explained by the different nature and mechanism of action of the two toxins. These results open new perspectives for comparative analysis of different BoNT serotypes which could have different effects on the muscle calcium read-out. An additional interesting possibility would be to test another serotype of BoNTs that cleaves a different protein than SNAP25, such as BoNT/B that cleaves VAMP protein, to compare the effect of different mechanisms of action.

Finally, the system described in our study could be optimized to be adapted for high-throughput screening. One possibility would be to use optogenetics reporter lines generated by stable transfection or genome editing as recently described [36]. This could reduce the variability related to several viral infections. Then, to support the fact that hMNs are well activated by light stimulation, we could use a genetic reporter of neuronal activity such as c-Fos to measure the hMNs activity [58, 59]. C-Fos is a proto-oncogene expressed within neurons following depolarization and can be identified by immunostaining. Its expression might be used as a marker for neuronal activity following stimulation [60]. Electrophysiology techniques could also be used to reinforce the hMNs activation during light stimulation. Another interesting approach that could be used would be to combine multi-electrode array recording and optogenetics and measure the electrical activity of the coculture system, that could be a new functional read-out to explore in a high-content context [36, 61, 62].

## Conclusions

Developing this human functional in vitro model system has potential to support studies in various scientific domains, from studying human physiology, investigating disease etiology and developing therapeutic design, to generating high throughput systems for drug screening [63]. In the context of BoNT studies, the generation of a muscle-nerve system based on hiPSC-derived technology sensitive to toxins provides the demonstration that an in vitro model of human functional motor endplate can provide major benefits in the production of preclinical data with high translational value for futures BoNT therapeutics.

## Supplementary Information

**Additional file 1: Figure S1.** hiPSCs WTSli020-B characterization and quality control. **a** Schematic representation of the culture and the amplification (manual and automated) of hiPSCs WTSli020-B. **b** Immunofluorescence analysis of common pluripotency markers (NANOG, OCT4, SOX2 and TRA1-60) in hiPSCs. Stainings were performed at P27 + 4. Scale bars: 50 μm. **c** Flow cytometry analysis of hiPSCs for TRA1-81 and SSEA4 pluripotency markers. Flow cytometry was performed at P27 + 8. **d** Detection of genomic abnormalities in hiPSCs. Samples collections were performed at P27 + 4.

**Additional file 2: Figure S2.** hMNs progenitors’ differentiation. **a** Schematic representation of the differentiation protocol based on Maury et al. (2015) to generate hMNs progenitors DIV 10. **b** Gene expression analysis in hMNs DIV 14 of relevant neuronal marker (MAP2), phenotypic markers (Islet1, AChE, ChAT), synaptic marker (SYP), and BoNT substrates (SNAP25, VAMP1, VAMP2, VAMP3) and receptors (SV2A, SV2B, SV2C, SYT1, SYT2). Expression is normalized to GAPDH and to control cDNA (human total adult spinal cord). **c** Immunofluorescence analysis of neuronal markers Islet1 and Tuj1 in hMNs DIV 14. Scale bars: 100 μm.

**Additional file 3: Figure S3**. Muscle-nerve coculture protocol. Immortalized human myoblasts were seeded in specific myogenic induction medium to induce their proliferation. Day1-post-plating myoblasts, hMNs progenitors DIV 10 previously obtained with the protocol developed by Maury et al. (2015) were seeded directly into myoblasts culture. The initial medium was switched with coculture medium to lead to the myotubes fusion, the hMNs differentiation, and the coculture maturation and survival until DIV 15.

**Additional file 4: Figure S4.** hMNs sensitivity to rBoNT/A in a single dose. **a** Representative Western blot showing the cleavage of SNAP25 protein from hMNs in coculture with myotubes treated with four doses of rBoNT/A (5 nM, 1 nM, 0.001 nM, 0.00001 nM) compared to toxin-free control dose (untreated). **b** Quantification of the SNAP25 cleavage for each dose. DIV 15 hMNs in coculture were exposed to rBoNT/A for 24 h before cell lysates were harvested, followed by Western blot to quantify SNAP25 cleavage. Data are represented as mean ± SEM (*n* = 3 independent experiment each performed in triplicate).

**Additional file 5: Figure S5.** Setup for optogenetic stimulation. **a** Myoblasts were transduced at DIV 1 with AAV2-CMV-GCaMP6f and hMNs were transduced at DIV 8 with AAV2-hSyn-ReaChR. The muscle-nerve coculture grew for two weeks and then to perform optogenetics assay. **b** (left) Quantification of the dose-dependent increase in AAV2-GFP expression in muscle cells. Data are represented as mean ± SD (*N* = 2 independent experiment, each performed in triplicate *n* = 3). (right) Immunofluorescence analysis of GFP^+^/Phalloidin^+^ muscle cells after AAV transduction at the selected MOI 5000 (represented by * in the graph). **c** Phase contrast images of muscle cells before transduction and day1-post-AAV2-CMV-GCaMP6f transduction (MOI 5000). Scale bars: 100 µm. **d** (left) Quantification of the dose-dependent increase in AAV2-GFP expression in hMNs. Data are represented as mean ± SD (*N* = 2 independent experiment, each performed in triplicate *n* = 3). (right) Immunofluorescence analysis of GFP^+^/Islet1^+^ hMNs after AAV transduction at the selected MOI 10 000 (represented by * in the graph). **e** Phase contrast images of hMNs before transduction and day2-post-AAV2-hSyn1-ReaChR transduction (MOI 10 000). Scale bars: 100 µm. **f** Relation between light power density and current density. **g** Setup for optical stimulation with a spinning disk microscope where the optical fiber is connected to a 590 nm laser connected to a channel controller.

**Additional file 6: Figure S6.** hMNs sensitivity to mBoNT. **a** EC_50_ curve for hMNs treated with serial doses of mBoNT for 24 h, compared to toxin-free control dose (untreated) using an antibody recognizing cleaved and uncleaved form of SNAP25 protein. DIV 15 hMNs were exposed to mBoNT for 24 h before cell lysates were harvested, followed by Western blot to quantify SNAP25 cleavage. Data are represented as mean ± SEM (*N* = 1 experiment performed in duplicate *n* = 2). **b** EC_50_ for hMNs is 0.01 pM (10^–14.14^).

**Additional file 7: Figure S7.** BoNTs effects on another line of motor neurons (56c2) connected to muscle cells and optogenetically controlled. Quantification of Ca^2+^ oscillations after rBoNT/A (5 nM or 0.0016 nM), mBoNT (5 nM or 0.0016 nM) or no BoNT exposure, before (pre-stim) and after (post-stim) red light stimulation over the time. Recordings were performed before treatment (basal), 4 h (H + 4) and 8 h (H + 8) after exposure of BoNTs or no treatment. Data are represented as mean ± SEM (*N* = 3). ANOVA with Sidak’s post hoc tests (****p < 0.0001; **p < 0.01; *p < 0.05; ns, not significant), each pre-stim recordings were compared to post-stim recordings.

**Additional file 8: Video S1.** Recording of myotubes contractions in cocultured myotubes.

**Additional file 9: Video S2.** Recording of myotubes contractions in mono-cultured myotubes. The myotubes did not contract in the absence of innervation with the motor neurons.

**Additional file 10: Video S3.** Recording of myotubes contractions in cocultured myotubes before and after tubocurarine treatment. Myotubes contractions were disrupted after the addition of tubocurarine.

**Additional file 11: Video S4.** Recording of myotubes contractions in cocultured myotubes before and after tetrodotoxin (TTX) treatment. Myotubes contractions were disrupted after the addition of TTX.

**Additional file 12: Video S5.** Recording of Ca^2+^ transient in cocultured myotubes before and after tubocurarine treatment. Ca^2+^ oscillations were disrupted after the addition of tubocurarine.

**Additional file 13: Video S6.** Recording of GCaMP6f^+^ myotubes signal before and after optogenetics stimulation with red light.

**Additional file 14: Video S7.** Effect of the addition of tubocurarine on the recording of GCaMP6f^+^ myotubes signal before and after optogenetics stimulation with red light.

## Data Availability

Not applicable.

## Abbreviations

AAV2: Adeno-associated virus serotype 2
AChE: Acetylcholinesterase
BDNF: Brain-derived growth factor
BoNTs: Botulinum neurotoxins
BSA: Bovine serum albumin
Ca^2^^+^: Calcium
ChAT: Choline acetyltransferase
CMV: Human cytomegalovirus
DAPI: 4',6-Diamidino-2-phénylindole
DAPT: γ-Secretase inhibitor
DIV: Day in vitro
DMEM: Dulbecco's modified eagle medium
DXT: Dexamethasone
EBs: Embryoid bodies
EC_50_: Half maximal effective concentration
EGF: Epidermal growth factor
EMA: European Medicines Agency
FBS: Fetal bovine serum
FDA: Food and Drugs Administration
FGF: Fibroblast growth factor
GDNF: Glial-derived neurotrophic factor
HBSS: Hanks' balanced salt solution
HC: Heavy chain
hiPSCs: Human-induced pluripotent stem cells
hMNs: HiPSC-derived motor neurons
hpe: Hour post-exposure
hPSCs: Human pluripotent stem cells
hSyn: Human synapsin 1
LC: Light chain
mBoNT: Modified botulinum neurotoxin
MF20: Myosin 4
MOI: Multiplicity of infection
PBS: Phosphate buffered saline
PenStrep: Penicillin/streptomycin
RA: Retinoic acid
rBoNT: Recombinant botulinum neurotoxin
ROI: Region of interest
SAA: Sarcomeric alpha actinin
SAG: Smoothened agonist
SMI32: Neurofilament H non-phosphorylated
SNAP25: Synaptosomal-associated protein 25KDa
SNARE: Soluble N-éthylmaleimide-sensitive-factor attachment protein Receptor
SV2: Synaptic vesicle protein 2
SYT: Synaptotagmin
TTX: Tetrodotoxin
VAMP: Vesicle-associated membrane protein
VTN-N: Vitronectin
β-ME: β-Mercaptoethanol
