# Supplementary figures and images for: Optogenetically controlled human functional motor endplate for testing botulinum neurotoxins
Source: Stem Cell Res Ther. 2021 Dec 5;12:599. doi: 10.1186/s13287-021-02665-3 (PMC8647380; doi:10.1186/s13287-021-02665-3)

a

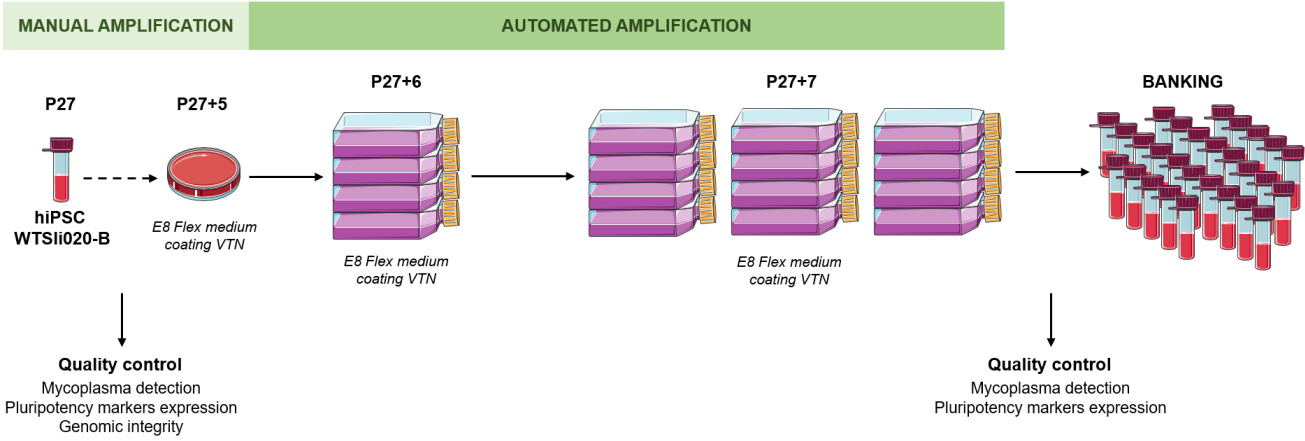

b

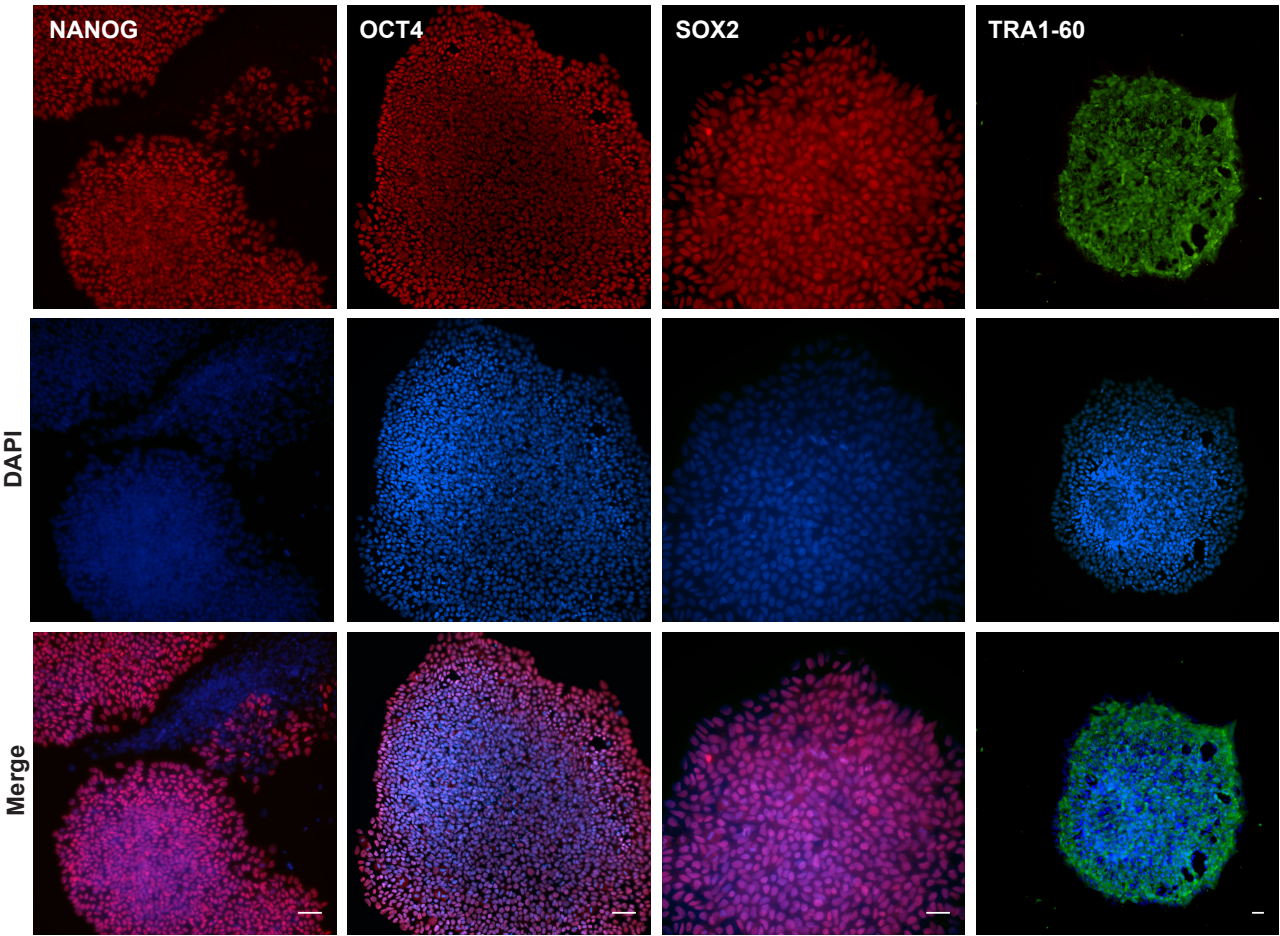

c

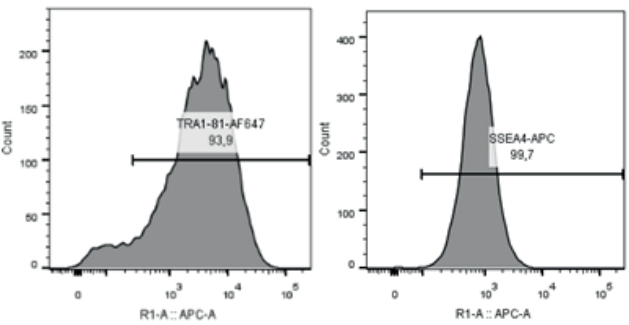

d

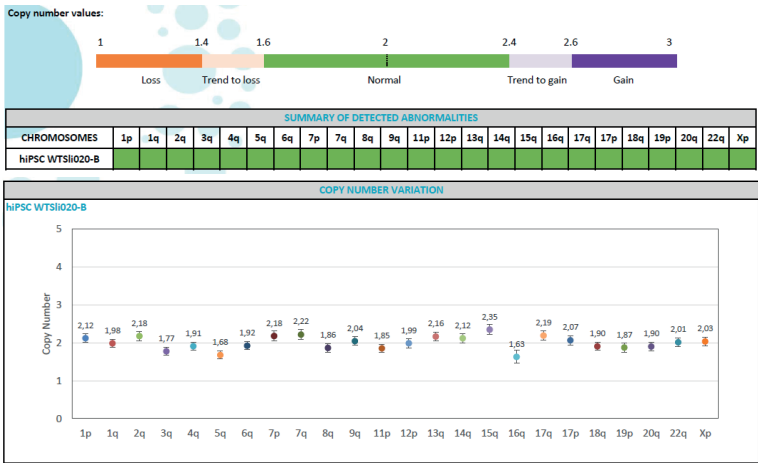

Supplement: Supplementary file 1 — Additional file 1: Figure S1. hiPSCs WTSli020-B characterization and quality control. a Schematic representation of the culture and the amplification (manual and automated) of hiPSCs WTSli020-B. b Immunofluorescence analysis of common pluripotency markers (NANOG, OCT4, SOX2 and TRA1-60) in hiPSCs. Stainings were performed at P27 + 4. Scale bars: 50 μm. c Flow cytometry analysis of hiPSCs for TRA1-81 and SSEA4 pluripotency markers. Flow cytometry was performed at P27 + 8. d Detection of genomic abnormalities in hiPSCs. Samples collections were performed at P27 + 4. [file 13287_2021_2665_MOESM1_ESM.pdf]

a

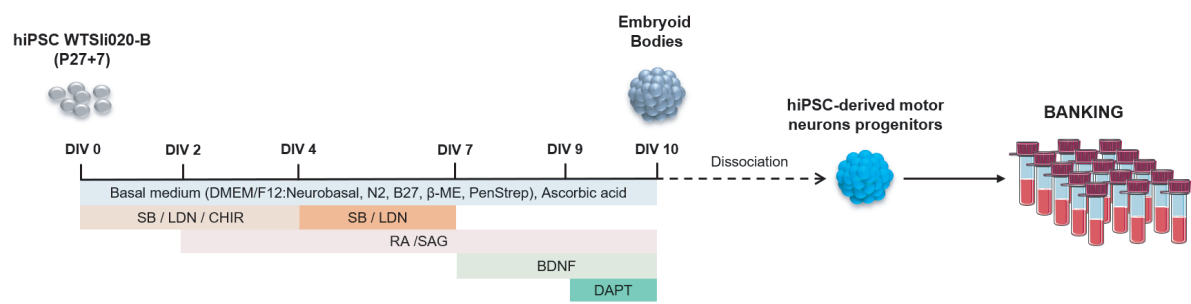

b

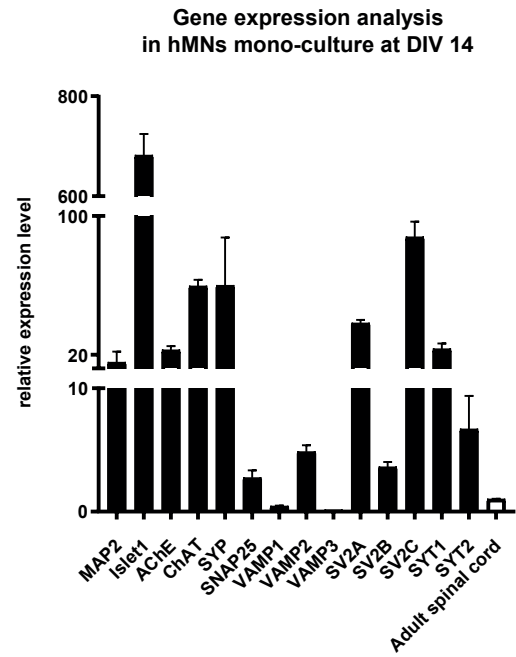

c

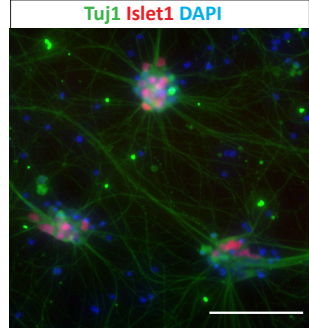

Supplement: Supplementary file 2 — Additional file 2: Figure S2. hMNs progenitors’ differentiation. a Schematic representation of the differentiation protocol based on Maury et al. (2015) to generate hMNs progenitors DIV 10. b Gene expression analysis in hMNs DIV 14 of relevant neuronal marker (MAP2), phenotypic markers (Islet1, AChE, ChAT), synaptic marker (SYP), and BoNT substrates (SNAP25, VAMP1, VAMP2, VAMP3) and receptors (SV2A, SV2B, SV2C, SYT1, SYT2). Expression is normalized to GAPDH and to control cDNA (human total adult spinal cord). c Immunofluorescence analysis of neuronal markers Islet1 and Tuj1 in hMNs DIV 14. Scale bars: 100 μm. [file 13287_2021_2665_MOESM2_ESM.pdf]

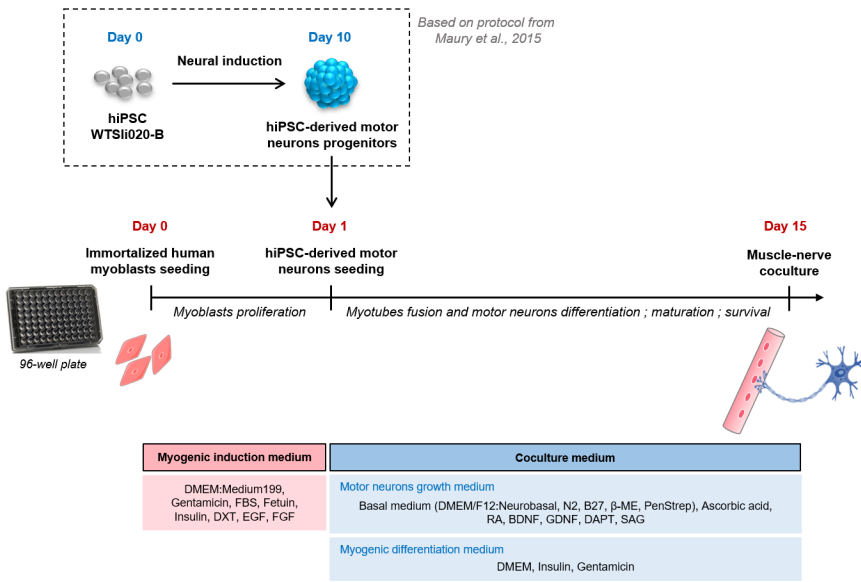

Supplement: Supplementary file 3 — Additional file 3: Figure S3. Muscle-nerve coculture protocol. Immortalized human myoblasts were seeded in specific myogenic induction medium to induce their proliferation. Day1-post-plating myoblasts, hMNs progenitors DIV 10 previously obtained with the protocol developed by Maury et al. (2015) were seeded directly into myoblasts culture. The initial medium was switched with coculture medium to lead to the myotubes fusion, the hMNs differentiation, and the coculture maturation and survival until DIV 15. [file 13287_2021_2665_MOESM3_ESM.pdf]

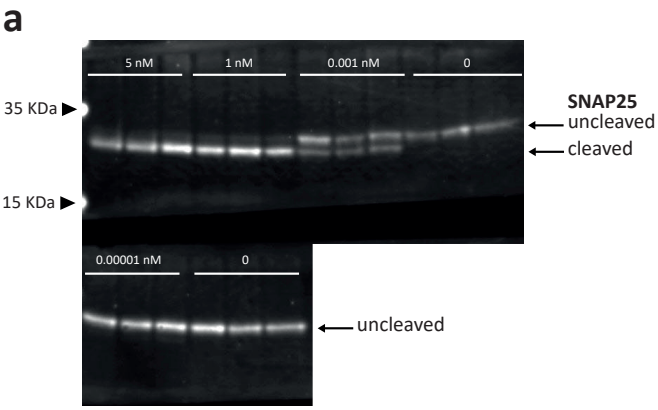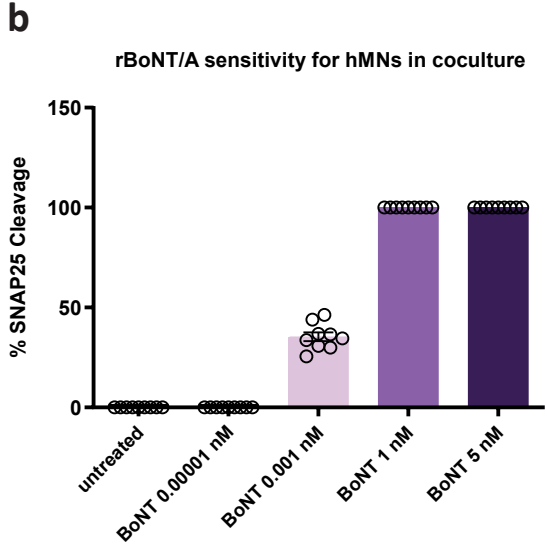

Supplement: Supplementary file 4 — Additional file 4: Figure S4. hMNs sensitivity to rBoNT/A in a single dose. a Representative Western blot showing the cleavage of SNAP25 protein from hMNs in coculture with myotubes treated with four doses of rBoNT/A (5 nM, 1 nM, 0.001 nM, 0.00001 nM) compared to toxin-free control dose (untreated). b Quantification of the SNAP25 cleavage for each dose. DIV 15 hMNs in coculture were exposed to rBoNT/A for 24 h before cell lysates were harvested, followed by Western blot to quantify SNAP25 cleavage. Data are represented as mean ± SEM (n = 3 independent experiment each performed in triplicate). [file 13287_2021_2665_MOESM4_ESM.pdf]

**a**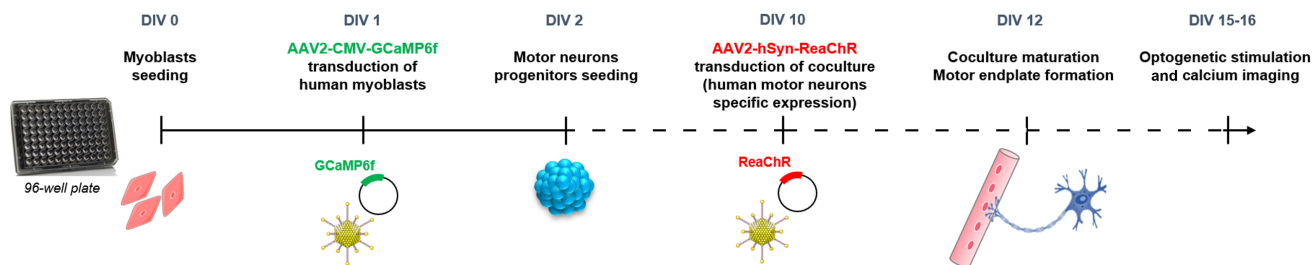**b**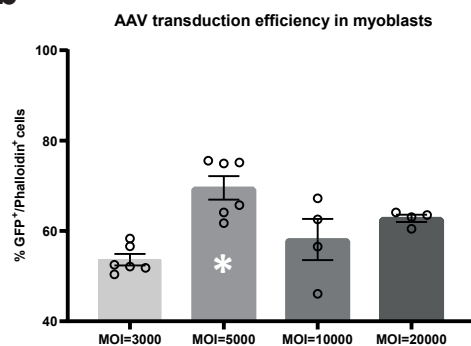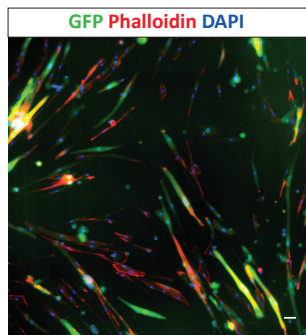**c**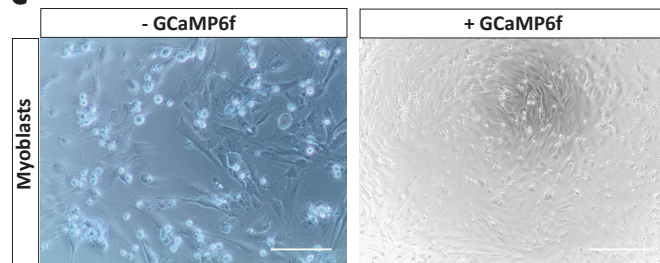**d**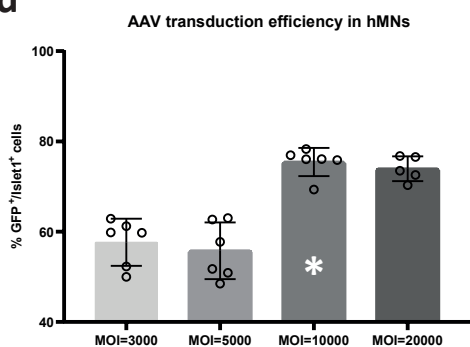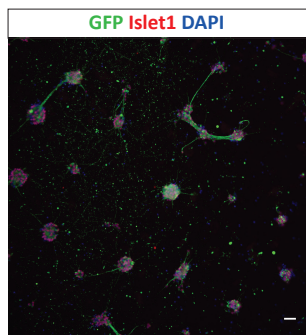**e**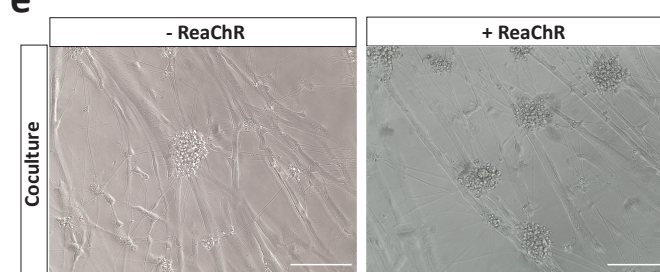**f**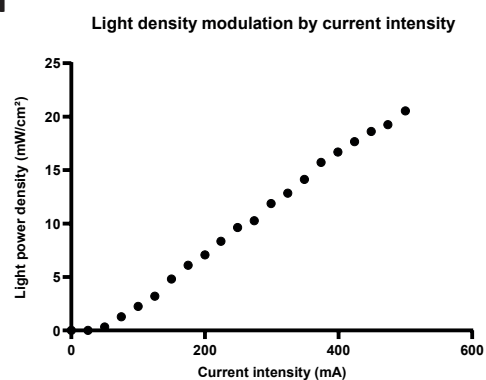**g**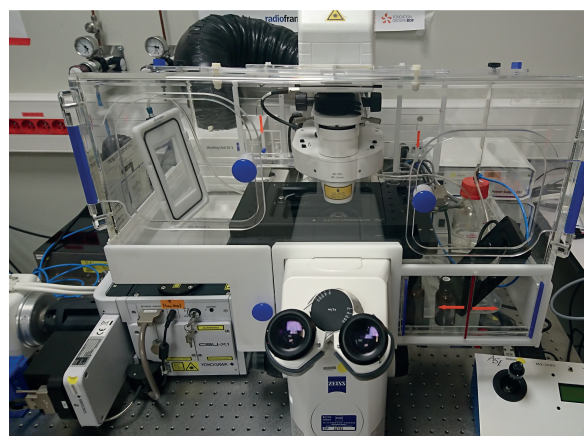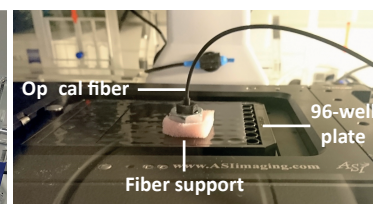

Op cal s mula on

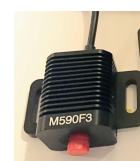

Laser 590nm

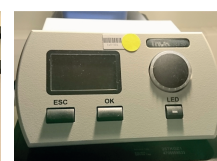

Channel controller

Supplement: Supplementary file 5 — Additional file 5: Figure S5. Setup for optogenetic stimulation. a Myoblasts were transduced at DIV 1 with AAV2-CMV-GCaMP6f and hMNs were transduced at DIV 8 with AAV2-hSyn-ReaChR. The muscle-nerve coculture grew for two weeks and then to perform optogenetics assay. b (left) Quantification of the dose-dependent increase in AAV2-GFP expression in muscle cells. Data are represented as mean ± SD (N = 2 independent experiment, each performed in triplicate n = 3). (right) Immunofluorescence analysis of GFP+/Phalloidin+ muscle cells after AAV transduction at the selected MOI 5000 (represented by * in the graph). c Phase contrast images of muscle cells before transduction and day1-post-AAV2-CMV-GCaMP6f transduction (MOI 5000). Scale bars: 100 µm. d (left) Quantification of the dose-dependent increase in AAV2-GFP expression in hMNs. Data are represented as mean ± SD (N = 2 independent experiment, each performed in triplicate n = 3). (right) Immunofluorescence analysis of GFP+/Islet1+ hMNs after AAV transduction at the selected MOI 10 000 (represented by * in the graph). e Phase contrast images of hMNs before transduction and day2-post-AAV2-hSyn1-ReaChR transduction (MOI 10 000). Scale bars: 100 µm. f Relation between light power density and current density. g Setup for optical stimulation with a spinning disk microscope where the optical fiber is connected to a 590 nm laser connected to a channel controller. [file 13287_2021_2665_MOESM5_ESM.pdf]

a

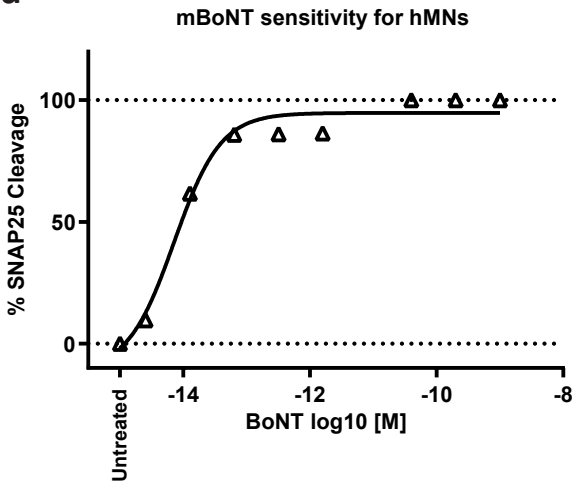

b

| <i>mBoNT</i> | hMNs in mono-culture |
|--------------|----------------------|
| Log EC50     | -14.14               |
| EC50 [pM]    | 0.01                 |

Supplement: Supplementary file 6 — Additional file 6: Figure S6. hMNs sensitivity to mBoNT. a EC50 curve for hMNs treated with serial doses of mBoNT for 24 h, compared to toxin-free control dose (untreated) using an antibody recognizing cleaved and uncleaved form of SNAP25 protein. DIV 15 hMNs were exposed to mBoNT for 24 h before cell lysates were harvested, followed by Western blot to quantify SNAP25 cleavage. Data are represented as mean ± SEM (N = 1 experiment performed in duplicate n = 2). b EC50 for hMNs is 0.01 pM (10–14.14). [file 13287_2021_2665_MOESM6_ESM.pdf]

GCaMP6f signaling after BoNT exposure

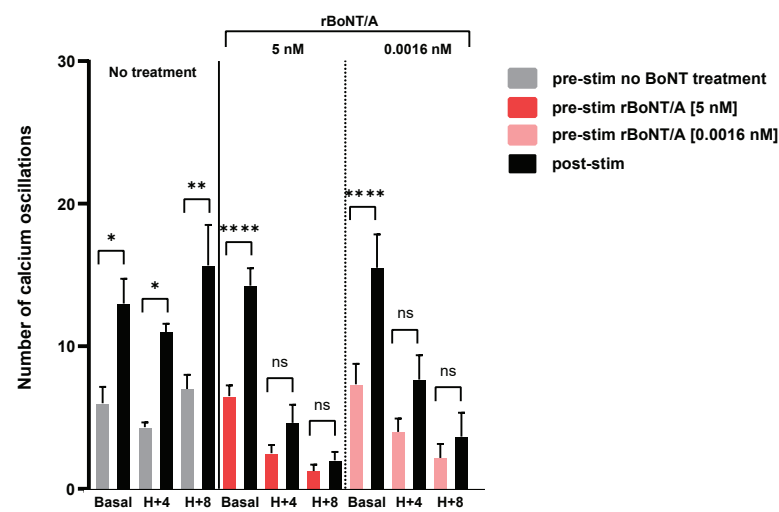

Supplement: Supplementary file 7 — Additional file 7: Figure S7. BoNTs effects on another line of motor neurons (56c2) connected to muscle cells and optogenetically controlled. Quantification of Ca2+ oscillations after rBoNT/A (5 nM or 0.0016 nM), mBoNT (5 nM or 0.0016 nM) or no BoNT exposure, before (pre-stim) and after (post-stim) red light stimulation over the time. Recordings were performed before treatment (basal), 4 h (H + 4) and 8 h (H + 8) after exposure of BoNTs or no treatment. Data are represented as mean ± SEM (N = 3). ANOVA with Sidak’s post hoc tests (****p < 0.0001; **p < 0.01; *p < 0.05; ns, not significant), each pre-stim recordings were compared to post-stim recordings. [file 13287_2021_2665_MOESM7_ESM.pdf]
